# In-Depth AGE and ALE Profiling of Human Albumin in Heart Failure: Ex Vivo Studies

**DOI:** 10.3390/antiox10030358

**Published:** 2021-02-27

**Authors:** Alessandra Altomare, Giovanna Baron, Marta Balbinot, Alessandro Pedretti, Beatrice Zoanni, Maura Brioschi, Piergiuseppe Agostoni, Marina Carini, Cristina Banfi, Giancarlo Aldini

**Affiliations:** 1 Department of Pharmaceutical Sciences (DISFARM), Università degli Studi di Milano, Via Mangiagalli 25, 20133 Milan, Italy; giovanna.baron@unimi.it (G.B.); marta.balbinot@studenti.unimi.it (M.B.); alessandro.pedretti@unimi.it (A.P.); marina.carini@unimi.it (M.C.); giancarlo.aldini@unimi.it (G.A.); 2 Centro Cardiologico Monzino, IRCCS, Via Parea 4, 20138 Milan, Italy; beatrice.zoanni@ccfm.it (B.Z.); maura.brioschi@cardiologicomonzino.it (M.B.); piergiuseppe.agostoni@cardiologicomonzino.it (P.A.); cristina.banfi@cardiologicomonzino.it (C.B.); 3 Dipartimento di Scienze Cliniche e di Comunità, Sezione Cardiovascolare, Università degli Studi di Milano, Via Festa del Perdono 7, 20122 Milan, Italy

**Keywords:** advanced glycation end-products (AGEs), advanced lipoxidation end-products (ALEs), albumin, heart failure, mass spectrometry

## Abstract

Advanced glycation end-products (AGEs) and advanced lipoxidation end-products (ALEs), particularly carboxymethyl-lysine (CML), have been largely proposed as factors involved in the establishment and progression of heart failure (HF). Despite this evidence, the current literature lacks the comprehensive identification and characterization of the plasma AGEs/ALEs involved in HF (untargeted approach). This work provides the first ex vivo high-resolution mass spectrometry (HR-MS) profiling of AGEs/ALEs occurring in human serum albumin (HSA), the most abundant protein in plasma, characterized by several nucleophilic sites and thus representing the main protein substrate for AGE/ALE formation. A set of AGE/ALE adducts in pooled HF-HSA samples was defined, and a semi-quantitative analysis was carried out in order to finally select those presenting in increased amounts in the HF samples with respect to the control condition. These adducts were statistically confirmed by monitoring their content in individual HF samples by applying a targeted approach. Selected AGEs/ALEs proved to be mostly CML derivatives on Lys residues (i.e., CML-Lys12, CML-Lys378, CML-Lys402), and one deoxy-fructosyl derivative on the Lys 389 (DFK-Lys 389). The nature of CML adducts was finally confirmed using immunological methods and in vitro production of such adducts further confirmed by mass spectrometry.

## 1. Introduction

Heart failure (HF) is the leading cause of morbidity and mortality in industrialized countries [1] and hence is considered a growing public health problem, primarily related to the aging population and the increasing incidence of HF in the elderly. HF is a complex clinical syndrome whose leading causes are myocardial infarction (MI), hypertension, cardiomyopathy, and valvular heart disease [2].

Over recent decades, evidence from experimental and clinical studies in humans and in animal models of HF have indubitably proved that oxidative stress is involved in the pathophysiology of congestive HF (CHF), contributing to the disease’s establishment and progression, especially when it is elevated systemically and in the myocardium [3]. A growing body of evidence suggests that in the setting of HF, reactive oxygen species (ROS) production within the myocardium and the vasculature is substantially increased [4]. Various sources of ROS, all interacting with each other, have indeed been identified, including mitochondria [5,6,7], NADPH oxidase, uncoupled endothelial nitric oxide synthase, and xanthine oxidase.

The oxidative stress condition alters lipid and sugar metabolisms, leading to the formation of several intermediates and breakdown products, the so-called reactive carbonyl species (RCS). RCS could exert a wide range of biological effects due to their ability to form, through non-enzymatic reactions, covalently bound adducts (Schiff base or Michael adducts) with nucleophilic groups of other macromolecules, such as nucleic acids, phospholipids, and proteins [4,8]. The process affecting proteins, namely the protein carbonylation process, leads to the formation of a heterogeneous class of compounds, which includes advanced glycation end-products (AGEs) and advanced lipoxidation end-products (ALEs) [9].

Circulating AGEs and ALEs, and, in particular, carboxymethyllysine (CML), a product adduct arising from glyoxal (deriving from both lipid and sugar oxidation pathways), have been proposed as factors involved in HF, being associated with the severity and the prognosis of the condition; furthermore, much evidence suggests their potential role in the onset of cardiovascular diseases (CVD) [8,10,11]. Further studies have demonstrated that AGEs, by stimulating the RAGE receptor, lead to prolonged cellular activation and the release of inflammatory cytokines, which, in turn, results in the development and progression of cardiac dysfunction and subsequent HF [10]. Besides the aforementioned CML, ALE products arising from malondialdehyde (MDA) and from the highly reactive unsaturated aldehydes (i.e., 4-hydroxynonenal (HNE) and acrolein (ACR)) have been the subject of in vitro and in vivo studies, all demonstrating a positive correlation between the toxic effects of HNE and acrolein and the particular sensitivity of cardiovascular tissues [12,13,14]. In particular, a study in animal models has demonstrated that chronic exposure to acrolein generates both blood and myocardial oxidative stress, and contextually protein adduct formation, thus suggesting that, following oral exposure, acrolein translocates via the circulation to the heart, where it modifies the cardiac proteins [15].

Despite these premises, a comprehensive identification and characterization of plasma AGEs and ALEs effectively involved in HF is lacking. Furthermore, the investigation of AGE/ALE adducts is still considered a very challenging issue because, from a chemical point of view, they are (i) quite heterogeneous, (ii) metabolically and chemically unstable, and (iii) contained in a negligible amount (nanomolar range) in respect to the native protein.

Recently, great effort has been devoted to the development of mass spectrometry methods for the determination of AGE/ALE levels in both tissue and blood samples, exploiting the potential of liquid chromatography–mass spectrometry (LC–MS), which is considered the most accurate technique available at the moment. However, untargeted LC–MS methods are not suitable for routine clinical use [8].

The aim of this work was to develop an analytical strategy to characterize in depth the AGE/ALE adducts with plasma albumin derived from patients with HF and from control subjects, with the final goal of optimizing a targeted approach that hopefully could be suitable for routine clinical use to discriminate pathological samples from healthy ones. Indeed, due to its abundance and relevant capability to scavenge several oxidants, forming covalent adducts with sugar/lipid oxidation electrophilic byproducts, albumin modifications might rise to the role of diagnostic and prognostic marker in cardiovascular diseases.

## 2. Materials and Methods

### 2.1. Reagents

Formic acid (FA), trifluoroacetic acid (TFA), and acetonitrile (ACN) were of LC–MS grade; tris(hydroxymethyl)aminomethane (Tris), sodium chloride (NaCl), sodium phosphate, sodium thiocyanate, ammonium bicarbonate, sodium borohydride (NaBH_4_), phosphate buffer saline (PBS, pH 7.4), Bradford reagent, fat dry milk, Tween 20, human serum from human male AB plasma, recombinant human serum albumin expressed in *P. Pastoris*, and all other chemicals were analytical grade and purchased from Sigma-Aldrich (Milan, Italy). Any KD™ Mini Protean^®^ TGX ™ precast gel, Standard Precision Plus pre-stained protein standards, Laemmli sample buffer (2 ×/4 ×), Running buffer, Bio-Safe Coomassie, PVDF membranes, threo-1,4-Dimercapto-2,3-butanediol (DTT), and iodoacetamide (IAA) were supplied by Bio-Rad Laboratories, Inc. (Hercules Contra Costa County, CA, USA). Anti-CML antibody (ab125145) and the goat anti-mouse IgG H&L–HRP (ab205719) were purchased from Abcam, Cambridge (U.K), while the SuperSignal West Atto Substrate was supplied by Thermo (Milan, Italy). Plasma levels of HbA1c were measured by ELISA (EIAlab, Antibodies-online). Ultrapure water was prepared with a Milli-Q purification system (Millipore, Bedford, MA, USA).

Plasma samples were obtained from a subset of 10 patients with chronic HF in stable conditions and from 8 age-matched healthy subjects (controls) from a previously enrolled population. The study was approved by the Ethical Committee, European Institute of Oncology and Monzino Cardiology Center (registration number R391/16-CCM406). The clinical characteristics of the subjects are shown in Tables S1 and S2.

### 2.2. Experimental Design

**Figure 1:**
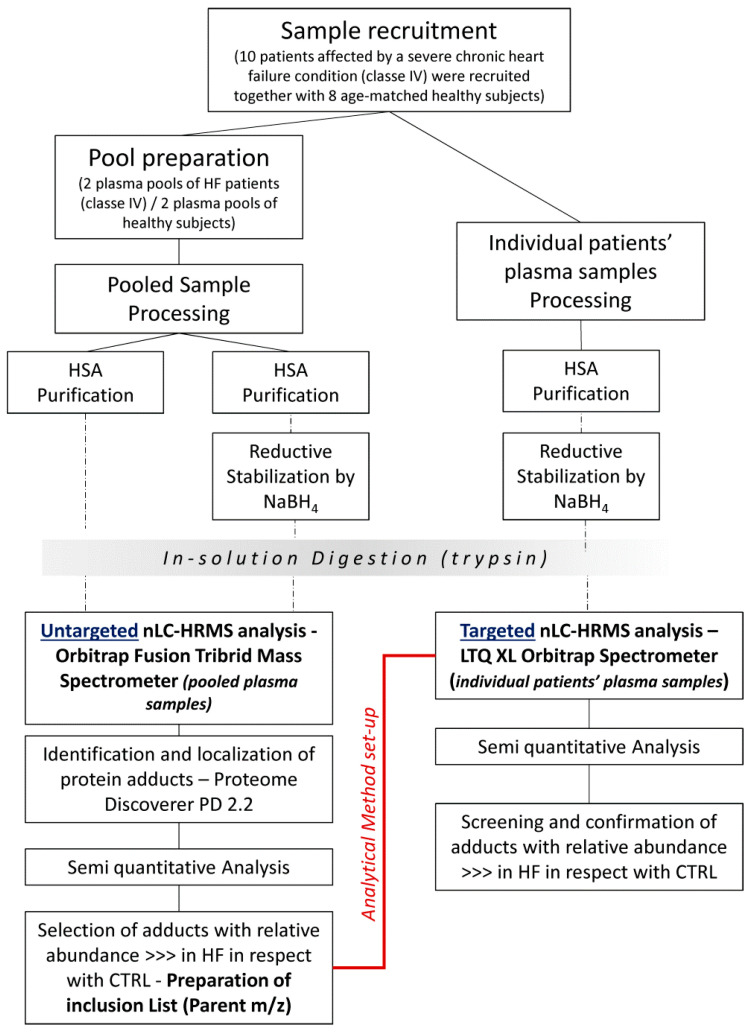
**Untargeted approach** for the detection of AGE/ALE protein adducts in HF and CTRL samples from a qualitative point of view, followed by a semi-quantitative comparison between the two conditions to identify those present in HF samples; MS Analyzer: Orbitrap Fusion Tribrid Mass Spectrometer. **Targeted approach** for the monitoring of candidate AGEs/ALEs adducts in individual subjects; MS Analyzer: LTQ ORBITRAP XL (Inclusion List).

The experimental workflow is shown in Figure 1. Briefly, both untargeted and targeted approaches were applied from a comprehensive characterization of the modifications occurring in human albumin (HSA) derived from HF patients and control (CTRL) subjects.

### 2.3. Sample Preparation

Albumin was purified from HF and CTRL samples by using the PierceTM Albumin depletion kit (Thermo Fisher Scientific Inc., Milan, Italy). Because of the high albumin concentration present in serum, each aliquot of 200 µL settled resin can bind sufficient albumin to process only 5–50 µL of serum sample, corresponding to 2 mg of HSA. In order to process larger serum sample volumes, two spin columns were used for each sample. Since HSA binding occurs at pH 6.0–9.0 and samples must be free of excess salt to ensure proper ionic strength, serum samples were desalted using desalting Amicon Filters, MWCO 10KDa (Millipore, Darmastadt, Germany) vs. a low-salt buffer (25 mM Tris, 75 mM NaCl, pH 7.5). Following serum desalting, the albumin purification by centrifugation was carried out according to the manufacturer’s instructions. The HSA elution fraction in 20 mM sodium phosphate, 250 mM sodium thiocyanate, pH 7.2 was further filtered on an Amicon Filter, MWCO 10 kDa (Millipore, Darmastadt, Germany) by rinsing with ammonium bicarbonate 50 mM, pH 8.5, to concentrate the protein and to prepare HSA samples in the optimum buffer conditions for the tryptic digestion. A rapid method for protein quantitation by Bradford assay was then performed on the retained HSA samples.

### 2.4. In Vitro Reductive Stabilization by NaBH_4_

The great potential of the mass analyzer Orbitrap Fusion was coupled with a sample preparation protocol based on reductive stabilization by NaBH_4_, which improves chemical stability and prevents the loss of Schiff base/Michael adducts by hydrolysis and retro-Michael addition reaction, respectively, during sample preparation as well as during collision-induced dissociation (CID), which otherwise will revert the adducts to the native condition. An aliquot of each purified HSA sample was diluted with 20 mM phosphate buffer saline (pH 7.4) to a final albumin concentration of 20 µM. Samples were incubated at room temperature for 60 min with NaBH_4_ (final concentration 5 mM) under the hood, and then desalted by Amicon Filter MWCO 10 kDa (Millipore), as above described, to remove NaBH_4_ excess. Bradford assay for protein quantification was then performed.

### 2.5. Protein Digestion

Non-reduced-HSA samples and NaBH_4_-reduced-HSA samples, both rinsed in ammonium bicarbonate 50 mM, were then subjected to tryptic digestion. Then, 50 µg of protein (in 30 µL of ammonium bicarbonate 50 mM) was incubated with 5 µL of dithiothreitol reducing solution (final conc.: 10 mM in 50 mM ammonium bicarbonate) at 56 °C for 1 hour and then with 5 µL of iodoacetamide alkylating solution (final conc.: 55 mM in 50 mM ammonium bicarbonate) at room temperature for 45 min in the dark. In-solution protein digestion was performed by adding 2 µg of sequencing-grade trypsin (Roche) dissolved in 10 µL of digestion buffer and incubating overnight. Peptide mixtures were extracted by using C18 ZIPTIP with a binding capacity of 2 µg each and eluted in 80% ACN, 0.1% FA. The extracts were then dried in a vacuum concentrator (Martin Christ, Osterode am Harz, Germany). Digested peptide mixtures were then dissolved in an appropriate volume (20 µL) of mobile phase for mass spectrometry (MS) analysis.

### 2.6. Untargeted nLC-HRMS Analysis—Orbitrap Fusion Tribrid Mass Spectrometer

To overcome the analytical limits of AGE/ALE adduct detection described above, an Orbitrap Fusion™ Tribrid™ Mass Spectrometer was selected as the MS analyzer due to the fact that it enables analysis of the most challenging low-abundance, high-complexity samples to identify more compounds faster, quantify more accurately, and elucidate structures more thoroughly.

Tryptic peptides dissolved in 0.1% FA were analyzed using a Dionex Ultimate 3000 nano-LC system (Sunnyvale, CA, USA) connected to an Orbitrap Fusion™ Tribrid™ Mass Spectrometer (Thermo Scientific, Bremen, Germany) equipped with a nano-electrospray ion source. Peptide mixtures were pre-concentrated onto an Acclaim PepMap 100–100 µm × 2 cm C18 and separated on an EASY-Spray column, 15 cm × 75 µm ID packed with Thermo Scientific Acclaim PepMap RSLC C18, 3 µm, 100 Å. The temperature was set to 35 °C and the flow rate was 300 nL/min. Mobile phases were the following: 0.1% formic acid (FA) in water (solvent A); 0.1% FA in water/acetonitrile (solvent B) with 2/8 ratio. Peptides were eluted from the column with the following gradient: 4% to 28% of B for 95 min and then 28% to 40% of B in 15 min, and to 95% within the following 6 min to rinse the column. Column was re-equilibrated for 25 min. Total run time was 145 min. One blank was run between triplicates to prevent sample carryover. MS spectra were collected over an *m*/*z* range of 375–1500 Da at 120,000 resolutions, operating in the data-dependent mode, cycle time 3 s between master scans. Higher-energy collisional dissociation (HCD) was performed with collision energy set at 35 eV. Each sample was analyzed in three technical replicates.

### 2.7. Data Processing

#### 2.7.1. Identification and Localization of Protein Adducts

The Proteome Discoverer software (PD, version 2.2.0.338, Thermo Scientific, USA), implemented with the SEQUEST algorithm, was used to compare the experimental full and tandem mass spectra with the theoretical ones obtained by the in silico digestion of the HSA sequence (Uniprot P02768). Trypsin was selected as the cleaving protease, allowing a maximum of 2 missed cleavages. Peptide and fragment ion tolerances were set to 5 ppm and 10 mmu, respectively. Cysteine carbamidomethylation was set as fixed modification (+57.02147), while methionine oxidation was allowed as a variable modification, along with a “state-of-the-art” set of AGE/ALE adduct mass shifts as listed in Table S3; in addition, for those adducts susceptible to reductive stabilization by NaBH_4_, a further set of mass shifts that considers the reduction step contribution (Table S3) was formulated. Since PD software allows searches against a restricted set of variable modifications, each MS raw file was reprocessed for all the processing workflows designed to cover a small set of protein adduct mass shifts, each grouped on the basis of the RCS generating the modification.

As a quality filter, only peptides with an XCorr value greater than 2.2 for doubly charged peptides, 2.5 for triply charged, 2.75 for quadruply charged peptide ions, and 3 for charge states quintuple or higher were considered as genuine peptide identifications. To ensure the lowest number of false positives, the mass values experimentally recorded were further processed through a combined search with the Database Decoy, where the protein sequences were inverted and randomized. This operation allowed the calculation of the false discovery rate (FDR) for each match, so that all the proteins out of range of FDR between to 0.01 (strict) and 0.05 (relaxed) were rejected.

For the localization of ALE-deriving modifications, the MS/MS spectra of modified peptides were manually inspected; for the confident mapping of the modification sites, spectra which matched the expected ions (b and/or y) neighboring the modified amino acid residue both at the N- and C-termini were requested.

#### 2.7.2. Semi Quantitative Analysis of AGE/ALE Protein Adducts on HSA

The extent of each protein adduct was determined by measuring the relative amount of the modified peptide in respect to the native one, by assuming that the ionization efficiencies of the native and the modified peptides were equal. In detail, the single ion traces (SIC) of the native and modified peptides were extracted from the total ion chromatogram (raw MS file) by setting as filter the *m*/*z* values of the corresponding precursor ions. The peak areas were then automatically measured with the dedicated Qual Browser tool in the Xcalibur data system (version 2.0.7, Thermo Scientific Inc., Milan, Italy) and then the relative abundance calculated by using Equation (1):

(1) $$Relative\;Abundance\;\%\;=\;\frac{Modified\;Peptide\;Peak\;Area}{\left(Modified\;Peptide\;Peak\;Area+Native\;Peptide\;Peak\;Area\right)}\;\times \;100$$

The relative abundance of each AGE/ALE protein adduct inspected was determined in both the HF-HSA and CTRL-HSA (reduced by NaBH_4_ and non-reduced) samples. 

Semiquantitative analysis was revealed to be crucial in the elucidation of the modifications (AGEs/ALEs) uniquely present or significantly more abundant in the pathological conditions with respect to the control. This in-depth inspection allowed the drawing up of a precursor inclusion list based on the *m*/*z* of the AGE/ALE adduct precursor ions selected, which was used to set up the advantageous targeted nLC-HRMS method.

### 2.8. Targeted nLC-HRMS Analysis of AGE/ALE Protein Adduct in HSA

Peptide mixtures from the in-solution digestion, processed as above reported, were separated by reversed-phase (RP) nanoscale capillary liquid chromatography (nanoLC) and analyzed by electrospray tandem mass spectrometry (ESI-MS/MS). For each analysis, 5 µL of solubilized peptides in 0.1% TFA was injected onto an Acclaim PepMap™ C18 column (75 µm x 15 cm, pores 100 Å, Thermo Scientific, Waltham, Massachusetts, USA), protected by a pre-column, namely the Acclaim PepMap™ (100 µm × 2 cm, pores 100 Å, Thermo Scientific, Waltham, Massachusetts, USA). Samples were loaded onto the pre-column by means of the loading pump at 5 µL/min of mobile phase consisting of 99% of buffer A-LP (0.1% TFA) and 1% of buffer B-LP (0.1% FA in ACN) for 3 minutes. After the loading valve switching, peptide separation was performed by the Nano Column Pump (NC-pump) with a 91-minute linear gradient of buffer B-NC-pump (0.1% FA in ACN) from 1% to 40%, and a further 12 minutes of linear gradient from 40% to 95% (Buffer B-NC-pump), 9 minutes at 95% of buffer B-NC-pump to rinse the column following the separative gradient, and the last 5 minutes served to re-equilibrate the column to initial conditions. The nano-chromatographic system, an UltiMate 3000 RSLC-nano System (Dionex), was connected to an LTQ-Orbitrap XL mass spectrometer (Thermo Scientific Inc., Milan, Italy) equipped with a Thermo Scientific dynamic Nanospray ion source set as follows: positive ion mode, spray voltage 1.7 Kv; capillary temperature 220 °C, capillary voltage 35 V; tube lens offset 120 V. The LTQ-Orbitrap XL mass spectrometer was operated in data-dependent acquisition mode (DDA) to acquire selected full MS spectra, whose parent *m*/*z* were listed in the parent mass list, and the corresponding MS/MS spectra. Full MS spectra were acquired in “profile” mode, by the Orbitrap (FT) analyzer, in a scanning range between 250 and 1500 *m*/*z*, using a capillary temperature of 220 °C, AGC target = 5 × 10^5^ and resolving power 30,000 (FWHM at 400 *m*/*z*). Tandem mass spectra MS/MS were acquired by the Linear Ion Trap (LTQ) in CID mode, automatically set to fragment the nine most intense ions in each full MS spectrum (exceeding 1x10^4^ counts) under the following conditions: centroid mode, normal mode, isolation width of the precursor ion of 2.5 *m*/*z*, AGC target 1x10^4^, and normalized collision energy of 35 eV. Dynamic exclusion was enabled (exclusion dynamics for 45 s for those ions observed 2 times in 10 s). Charge state screening and monoisotopic precursor selection were enabled, and singly and unassigned charged ions were not fragmented. Xcalibur software (version 2.0.7, Thermo Scientific Inc., Milan, Italy) was used to control the mass spectrometer.

Semiquantitative analysis was performed for MS/MS spectra corresponding to AGE/ALE adducts whose fragmentation was manually checked, comparing the experimental fragmentation pattern with theoretical ones obtained by using the peptide tool implemented in the Molecular Weight Calculator software (version 6.50–Build 246; Freeware, by Matthew Monroe). The relative abundance values were calculated by using Equation (1); this final operation allowed an in-depth screening of the selected modifications from pooled plasma samples, moving on to individual patients’ plasma samples.

### 2.9. Detection of CML-Adducted Protein by Western Blot

#### 2.9.1. In Vitro HSA-AGE/ALE Production

HSA modified with glyoxal (GO) was prepared by dissolving commercial recombinant HSA from Sigma in 10 mM phosphate buffer pH 7.4 at a concentration equal to 100 μM (6.7 mg/mL). HSA was incubated in the dark at 37 °C and 400 rpm and using the 1:10 and 1:100 molar ratios between protein and GO. The reactions were stopped after 48 h by removing the excess of RCS by ultrafiltration against water using Amicon Ultra filter units 0.5 mL, cut-off 10 kDa (Millipore). HSA adduct formation was confirmed by SDS-PAGE by loading 13.4 µg for each sample.

#### 2.9.2. Western Blot

For the immunoblot analysis, 0.75 µg of HSA-AGEs/ALEs standard and around 1.2 µg of plasma proteins taken from healthy subjects (CTRL samples) and heart-failure-affected patients (HF samples) were separated by SDS-PAGE in reducing conditions; to 1 µL of the diluted protein samples in water, 5 µL of Laemmli Sample Buffer 4x and 14 µL of 71 mM DTT were added. The samples were denatured, incubating for 5 min at 95 °C. CTRL and HF samples were separated on Any KD ™ Mini Protean^®^ TGX ™ precast gels. Proteins were then transferred onto PVDF membranes using the TranBlot Turbo Transfer System Bio-Rad (BioRad).

Blotted membranes were incubated in Ponceau S Staining Solution (0.5% *w/v* in 1% *v/v* acetic acid) for 1 min at room temperature, and then washed in water until distinct reddish-pink protein bands were visible (1–5 min). Images were acquired by means of the ChemiDoc MP Imaging System (Bio-Rad, Hercules, CA, USA) and subsequently washed in 1x Tris-buffered saline (TBS) with 0.2% Tween (TBST) several times for 5 min at room temperature until protein bands were no longer visible.

After incubation of the blotted membranes with 5% fat dried milk in TBST overnight at 4 °C, the membrane was washed 10 times (5 min) in TBST and then incubated for 1 h at room temperature with primary antibody, mouse monoclonal anti-CML antibody (1:2000, ab125145, Abcam, Cambridge, UK). The PVDF sheet was washed as previously described and then incubated with secondary antibody, goat anti-mouse IgG H&L–HRP (1:20000, ab205719, Abcam, Cambridge, UK) for 1 h. After 10 washing steps in TBST, chemiluminescence detection was performed using SuperSignal West Atto Substrate (Thermo) at room temperature; ECL Western Blot detection reagent was added for 30 s, and the signal was acquired (3-sec exposure in ChemHighsensitivity mode), placing the blotting membrane back on the sample stage of the ChemiDoc MP Imaging System (Bio-Rad, Hercules, CA, USA) and elaborating the image using Image Lab 4.1 software (Bio-Rad, Hercules, CA, USA).

### 2.10. Measurement of CML-Adducted Protein Content by ELISA

Nε-(Carboxymethyl)lysine (CML) protein adducts in plasma (CTRL and HF samples) were measured using a commercially available ELISA kit (OxiSelect Nε-(carboxymethyl)lysine (CML) Competitive ELISA Kit, Cell Biolabs, Inc.; Valter Occhiena S.R.L., Torino, Italy). Plasma was diluted in PBS up to 1 µg/mL as final protein concentration and tested in triplicate according to the manufacturer’s instructions; the absorbance was read at 450 nm on a PowerWave HT microplate reader (BioTek, Winooski, VT, USA).

### 2.11. In Vitro AGE/ALE-HSA Adduct Confirmation by Mass Spectrometry

#### 2.11.1. In Vitro AGEs/ALE Production

Commercial recombinant HSA modified with glyoxal (GO) was prepared by dissolving HSA in 10 mM phosphate buffer, pH 7.4, at a concentration equal to 100 μM (6.7 mg/mL). HSA was incubated in the dark at 37 °C and 400 rpm and using the 1:10, 1:100, 1:1000 molar ratios between protein and GO. In parallel, plasma (Sigma) was incubated in the dark at 37 °C and at 400 rpm in molar ratios protein (HSA, 600 µM):GO equal to 1:100, 1:1000. HSA incubated without RCS was used as a control untreated sample. The reactions were stopped after 48 and 72 h, removing the excess of RCS by ultrafiltration against water using Amicon Ultra filter units 0.5 mL, cut-off 10 kDa (Millipore). HSA adduct formation was confirmed by SDS-PAGE by loading 13.4 µg for each sample.

#### 2.11.2. Protein Digestion (HF Samples and In Vitro AGEs/ALEs) and MS Analysis

On the basis of the measured protein concentration, in vitro prepared samples (HSA/plasma–GO) and the 2 patient plasma samples (HF57–HF114) were diluted up to 3 µg/µL in ammonium bicarbonate 50 mM. Protein digestion and peptide extraction by using ZIPTIP C18 was performed as previously described. Dried peptide mixtures were then dissolved in 0.1% TFA and 5 µL of each sample was injected twice on the analytical platform set as specified for the targeted nLC-HRMS analysis of AGE/ALE protein adduct in HSA, except for the parent mass list (not included); in essence, the LTQ-Orbitrap XL mass spectrometer was operated in the canonical data-dependent acquisition mode (DDA) to acquire selected full MS spectra and the corresponding MS/MS spectra.

Full and tandem mass spectra (raw data) were manually inspected with the aim of confirming the correspondence of both the theoretical isotopic and the fragmentation patterns, each derived from the in-depth investigation of protein adducts characteristic of the pathological condition (HF).

## 3. Results

### 3.1. Untargeted nLC-HRMS Analysis of AGE/ALE Modifications in HSA

The untargeted approach was used to detect characteristic HSA AGE/ALE protein adducts in HF patients by means of qualitative analysis followed by a semi-quantitative comparison between HF and CTRL samples. To overcome the analytical limits regarding AGE/ALE adduct identification, the great potential of the Orbitrap Fusion Tribrid Mass analyzer was coupled to the reductive PTMs’ stabilization by NaBH_4_ finalized to stabilize Schiff base/Michael adducts.

**Figure 2:**
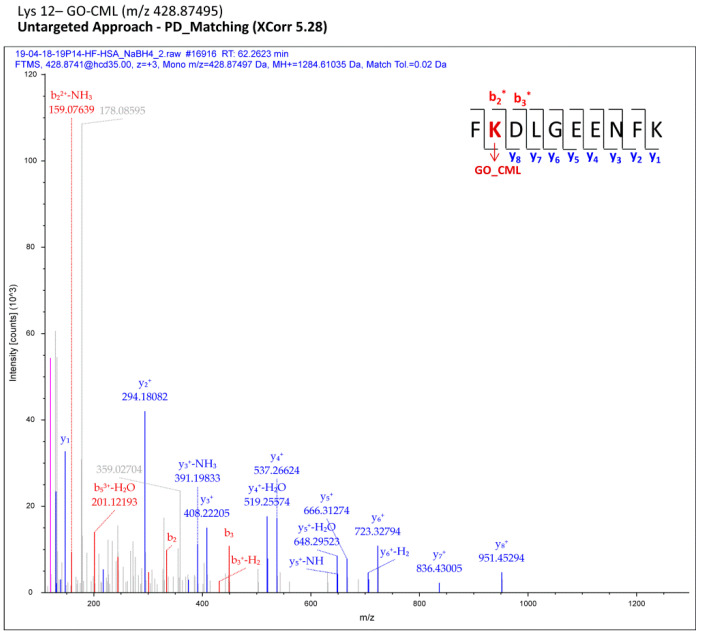
Fragmentation spectra related to a carboxymethyl-Lys adduct (CM-Lys 12) identified through the computational analysis (Proteome Discoverer) and confirmed by manual check. (*modified fragment ion)

As described in the Section 2, each MS raw file was reprocessed for all the processing workflows (Proteome Discoverer), each designed to cover a small set of protein adduct mass shifts; all the assignments computationally regarded as reliable were finally merged in a comprehensive list for each sample (pooled CTRL/pooled HF). Filtered MS/MS spectra of modified peptides were manually inspected with the aim of selecting only the most confident adducts and of localizing AGE/ALE adduct sites. As an example, Figure 2 shows a fragmentation spectrum related to one adduct identified through the computational analysis (Proteome Discoverer) and confirmed by manual check, namely the carboxymethyl-Lys adduct (CM-Lys 12).

In this way, we globally identified several AGE/ALE adducts on HSA (without distinguishing between CTRL and HF samples) mainly attributable to carboxymethyl derivatives and acrolein Michael adducts on Lys/His residues (Table 1).

**Figure 3:**
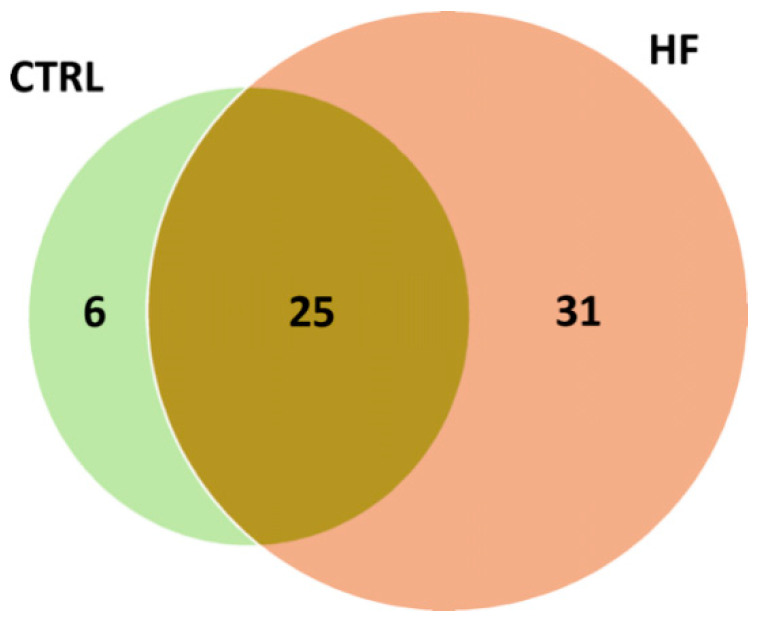
Venn diagram depicting the distribution of putative adducts in the two conditions (CTRL vs. HF).

The distribution of putative adducts in the two conditions is depicted in Figure 3. In total, 31 AGE/ALE adducts were uniquely identified in HSA derived from HF patients (HF-HSA), six were detected only in HSA from control subjects (CTRL-HSA), whilst 25 were found in both groups.

A semi-quantitative analysis was carried out by profiling the molecular ion of the peptide containing the AGE/ALE modification as well as that of the native one. The extent of each protein adduct was determined by measuring the relative amount of the modified peptide with respect to the native one by using Equation (1), as above described (Figure S1, example of the semi-quantitative analysis). The relative abundance of each AGE/ALE protein adduct inspected was determined in both the HF-HSA and CTRL-HSA (reduced by NaBH_4_ and non-reduced) samples and the results were finally merged.

Acrolein adducts were found to be characterized by an unexpected, very high percentage in HF, more than 30%. We found that this extensive modification was due to an overestimation caused by the covalent modification of His residues by iodoacetamde forming the corresponding carbamidomethylated derivatives whose isotopic mass (C12 *m*/*z* 57.02092; C13 +1 *m*/*z* 58.02427) overlapped the monoisotopic mass of the ACR adduct in reduced form (C12 *m*/*z* 58.00493). The reason that a higher extension of carbamidomtheylation of albumin from HF in respect to the control occurs needs to be clarified. For this reason, ACR adducts were excluded.

Moreover, manual inspection of the spectra in terms of (i) overlapping fragmentation patterns, (ii) signal intensity of the corresponding fragments (number of counts), (iii) precursor isotope pattern check, and (iv) retention time drastically reduced the number of modifications previously identified by means of automated qualitative analysis using Proteome Discoverer software. Analysis using bioinformatics software should be considered only as a preliminary step that helps the operator to screen the whole range of possible modifications (untargeted approach), but it is not sufficient in defining accurately the modifications that need to be thoroughly investigated.

Table 2 shows the protein adducts identified and the corresponding relative abundance values (%) calculated, consisting of only those modifications (AGEs/ALEs) uniquely present or significantly more abundant in the pathological conditions with respect to the control. Those modifications present to a similar extent in both the CTRL-HSA and HF-HSA were also considered to better investigate their nature through the targeted analysis of individual patients’ samples. Furthermore, equal content of some modifications (i.e., carboxymethyl derivatives) was detected in the NaBH_4_-reducing conditions and in the non-reducing conditions. We therefore adopted the reducing conditions for the targeted analysis in order to reduce the number of samples to be analyzed in MS.

**Figure 4:**
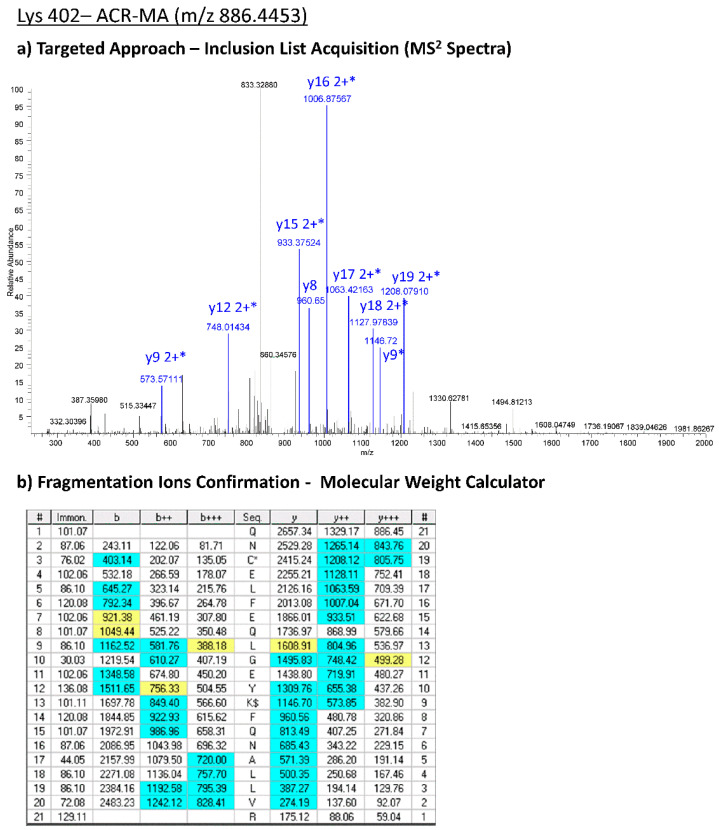
Experimental MS^2^ spectra acquired in a targeted way corresponding to the CML-Lys 402 panel (**a**), and the corresponding theoretical fragmentation pattern obtained by means of Molecular Weight Calculator software panel (**b**). (*modified fragment ion).

This detailed inspection allowed the drawing up of a precursor inclusion list (Table S4) based on the *m*/*z* of the AGE/ALE adduct precursor ions selected and the corresponding native ones thus used to set up the advantageous targeted-nLC-HRMS method in order to statistically confirm those characteristic protein adducts, by monitoring them in single patients affected by cardiovascular diseases. Since, for the target analysis, we moved to a different mass spectrometer (LTQ-Orbitrap XL), the inclusion list method was previously tested on pooled samples (HF-HSA/CTRL-HSA) in order to confirm the strength and reproducibility of the method. By manually checking the fragmentation patterns, all the modifications identified by using the Orbitrap Fusion Tribrid mass analyzer (shown in Table 2) were confirmed by applying the targeted approach on the LTQ-Orbitrab XL. As an example, in Figure 4, the experimental MS2 spectra acquired by means of the targeted approach corresponding to the CML-Lys 402 are reported.

Overall, the first part of the work (untargeted approach) allowed the identification, by analyzing pooled plasma samples, of albumin AGEs/ALEs characteristic of the pathological condition (HF); these adducts were then monitored in a population of HF single patients to be validated.

### 3.2. Targeted HR-MS Profiling of AGEs and ALEs (Single Patient Investigation)

The targeted approach was aimed at confirming the uniqueness of the candidate HF-specific AGE/ALE adducts selected by means of the untargeted approach, monitoring them in a population of individual HF patients’ plasma samples.

**Figure 5:**
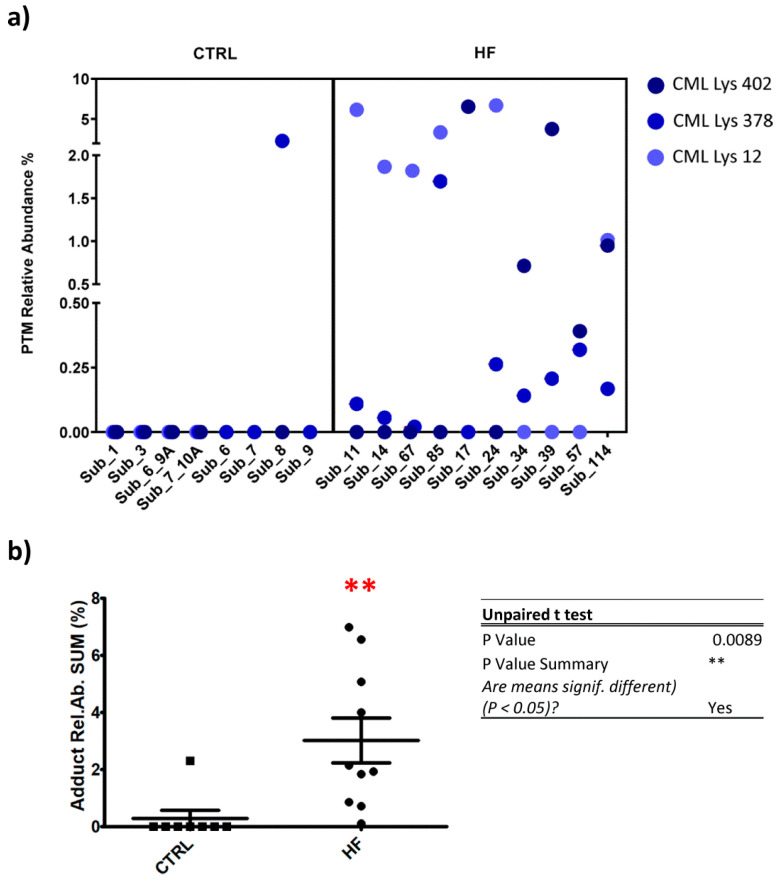
Panel (**a**): Graphical representation of the extent of the most representative AGE/ALE adducts in both CTRL and HF individual subjects. The color code (see legend) serves to distinguish each CML protein adduct. Panel (**b**): Plot showing the distribution of the sum of the CML relative abundances calculated for each subject (CTRL vs. HF); the difference between the two groups was proven to be significant by means of an unpaired *t*-test (*p* < 0.01). (** *p*-value summary).

Semi-quantitative analysis in single patients’ samples was performed for those AGE/ALE adducts whose retention time values corresponded to the recorded ones in experiments carried out using the untargeted approach and whose MS/MS spectra overlapped the theoretical fragmentation pattern obtained by using the “peptide tool” implemented in the Molecular Weight Calculator software. The relative abundance values were calculated by using Equation (1), as reported in the Section 2; this final operation allowed an in-depth screening of the selected modifications from pooled plasma samples, moving in the single patient plasma samples. Figure 5 depicts the relative content of the most representative protein adducts detected in the single patient analysis list, among which only four proved to be significantly more abundant in pathological conditions with respect to the control.

As above outlined, the targeted approach was set to search for not only those adducts showing a uniqueness in HF samples, but also those detected, albeit to a lesser extent, in CTRL samples.

For these adducts also identified in CTRL samples, a t-test (and nonparametric test) was performed to evaluate the significance of mean value differences (*p* < 0.05): control condition vs. pathological. one of these adducts revealed a HF-relative abundance (mean) or CTRL-relative abundance (mean) that was significantly different.

The remaining CML-Lys 12, CML-Lys 402, CML-Lys 378 were found under the LLOD (lower limit of detection) in CTRL groups, except for CML Lys 389 in subject 8, while at least one of these adducts was detectable in all the HR subjects and two in 9 out of 10 cases (Figure 5, panel a). Figure 5 panel b shows a plot in which, for each subject, the sum of the CML relative abundances is reported and a significant difference between CTRL and HF group was found (*p* < 0.01). DFK-Lys 389 was also found to be increased in HF subjects in respect to the CTRL.

### 3.3. Detection and Content Measurement of CML-Adducted Protein

With the aim of confirming HSA as the plasma protein effectively carrying CML adducts, an anti-CML Western Blot was performed on plasma samples of a subgroup of CTRL and HF samples.

**Figure 6:**
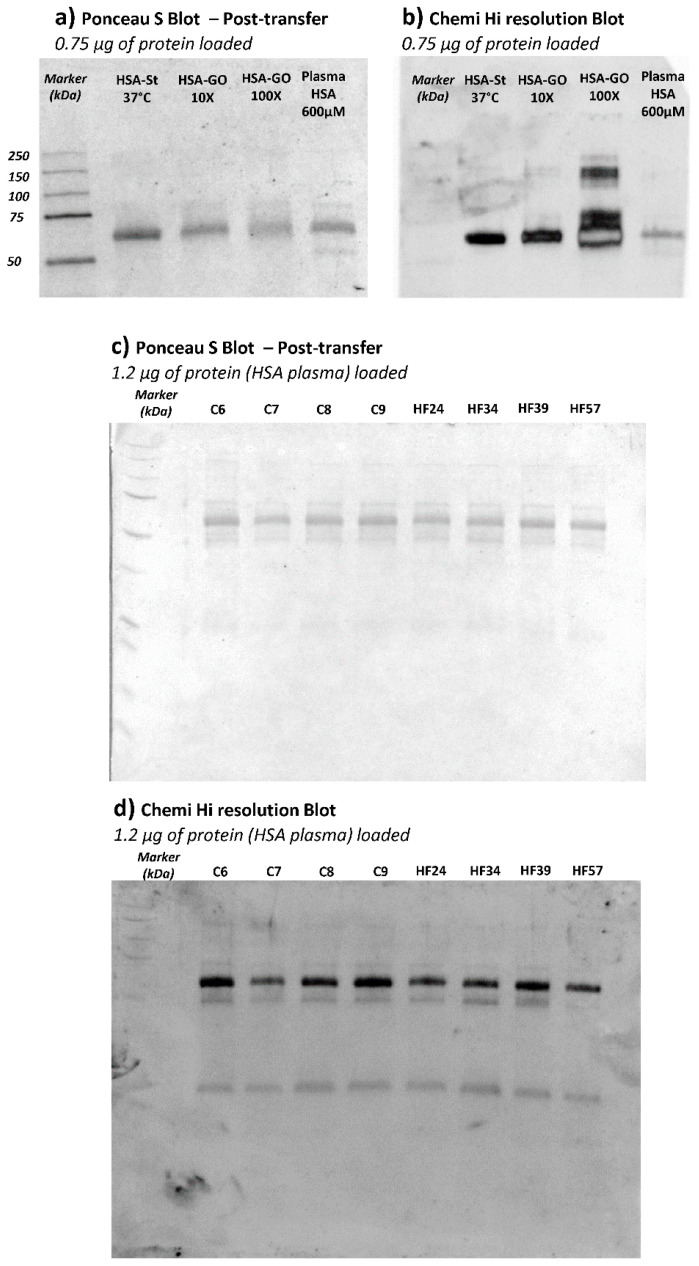
Ponceau S-stained blotted membrane of HSA AGE/ALE standards (HSA incubated at 37 °C, HSA incubated with glyoxal at 1:10 and 1:100 molar ratio), panel (**a**); Anti-CML chemi-blot membrane (HSA AGE/ALE standards) acquired after 3 sec of exposure in high-resolution mode, panel (**b**); Ponceau S-stained blotted membrane of four CTRL plasma samples (C 6-7-8-9) together with four HF plasma samples (HF 24-34-39-57), panel (**c**); Anti-CML chemi-blot membrane (CTRL/HF plasma samples) acquired after 3 sec of exposure in high-resolution mode, panel (**d**).

Figure 6 (panels a and b) shows the Ponceau stained and the corresponding anti-CML immunoblotted membrane of HSA and HSA incubated with GO. This analysis served to confirm the glyoxal dose-dependence effect in terms of CML adduct formation on HSA. For the test, we used HSA from Sigma which was isolated from a pool of human plasma and lyophilized; by an internal quality control study carried out in our laboratory, we found that this commercial protein, in respect to native HSA isolated from fresh human plasma, is characterized by a significant content of AGEs and ALEs, including CML adducts, as identified by a bottom-up MS approach (data not shown). Such modifications are in line with the significantly positive CML response as depicted in Figure 6, panel B; in any case, we did nevertheless observe a further increase in band intensity in relation to GO incubation which increased dose-dependently.

Figure 6, panel c depicts the Ponceau-stained membrane, showing the plasma protein profile and the corresponding anti-CML developed through immunoblotting (panel d). Although the anti-CML antibody recognized more than one band along with the plasma profile, the most intense one was that detected at around 66 kDa, attributed to the HSA; the other two bands of around 50 and 25 kDa detected by immunoblot could be related to the reduced subunit of immunoglobulins. This evidence could prove the scavenging ability of HSA against circulating RCSs, even in physiological conditions, since no remarkable differences in terms of intensity are inferable from the bands among the samples loaded. Through the application of immunoblotting, we can see that the HSA is the lead plasma protein susceptible to glyoxal reactivity. The results were then validated by repeating the experiments by using HSA isolated from the plasma samples. The CML positive bands detected in HSA samples were found superimposable on those found in plasma samples (data not shown). Despite its reasonable sensitivity, unlike the MS approach, the Western Blot technique was not able to detect differences between CTRL and HF groups and this was clearly due to the differences in the accuracy, precision, and selectivity of the two methods. Nevertheless, the WB approach was used in this study to profile the main plasma proteins affected by CML, revealing HSA to be the main protein modified by GO. The MS method was then found to be able to detect differences in semi-quantitative terms.

Nε-(Carboxymethyl)lysine (CML) protein adducts in plasma (CTRL and HF samples) were measured using a commercial competitive ELISA Kit; the kit is designed to outline the CML protein content expressed as a concentration value (ng CML-prot/mL); 10 HF plasma samples and 7 CTRL samples were measured in triplicate.

**Figure 7:**
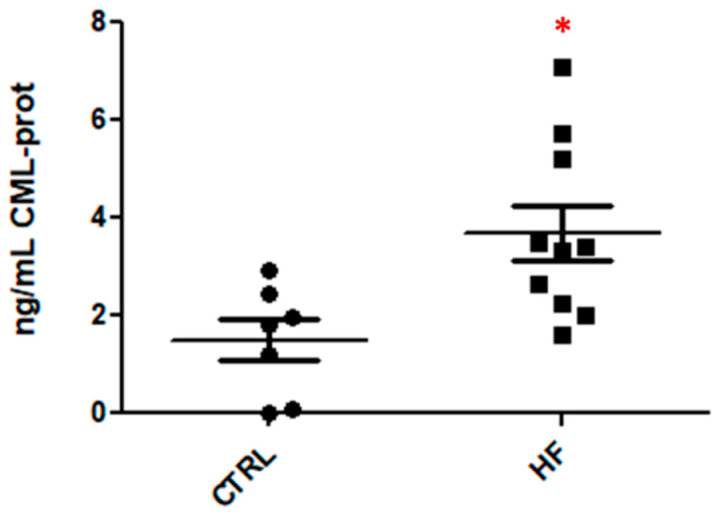
Measurement of CML-adducted protein content by ELISA; Statistical analysis: unpaired *t*-test (*p* value 0.0122). (* p-value summary).

The repeated measures relative to the two groups are represented by vertical scatter plots showing the distribution of all the samples (Figure 7). Data were analyzed with GraphPad Prism v5.03 using the unpaired t-tests; a *p* value of 0.0122 (<0.05) was considered, confirming our assumptions made on the basis of the MS results.

An ELISA test of HNE protein adducts was then carried out since the qualitative analysis (untargeted approach) identified HNE adducts in CTRL and HF samples. The results did not show any significant difference (data not shown), thus confirming the semi-quantitative analysis performed by MS approach.

### 3.4. AGE/ALE HSA Adduct Confirmation by Mass Spectrometry

As above described, HSA modified with glyoxal (GO), intended as a “reference material”, was prepared at increasing molar ratios between protein and GO (1:10, 1:100, 1:1000). Besides the molar ratio, the time-dependence effect was evaluated by stopping the reaction at two time points: after 48 and 72 h. The HSA adduct formation was confirmed by SDS-PAGE (Figure S2); HSA modified by GO is characterized by a different migration pattern on the gel, with the appearance of oligomeric bands proportional to the increase in the HSA–GO molar ratio: as the molar ratios increased, a predominance of high-molecular-weight species (HMW) was observed, attributed to the RCS protein adduct. The incubation time, in essence, did not significantly affect the nature of the protein profile.

In vitro prepared samples (HSA/plasma–GO) and two patient plasma samples (HF57-HF114) were injected twice on the analytical platform operating in the canonical data-dependent acquisition mode (DDA) to acquire selected full MS spectra and the corresponding MS/MS spectra with the aim of confirming the formation of such modifications described in the ex vivo studies.

Overall, from a qualitative point of view, the manual inspection of the full and tandem mass spectra confirmed the correspondence of both the theoretical isotopic and the fragmentation patterns of such adducts selected as protein adducts to be characteristic of the pathological condition (HF); also, the retention time was checked. Table 3 summarizes the presence/absence of the specific species (CML adducts) based on the fragmentation profile and retention time in the most representative samples.

## 4. Discussion

The present paper reports the set-up of an analytical platform for the identification of plasma AGEs/ALEs using an untargeted approach and its application in the identification of albumin adducts in the plasma of HF patients.

As stated in the Introduction, the literature lacks an in-depth and thorough description of AGE/ALE adducts characteristic of a pathological condition related to cardiovascular risk; the evidence published so far is mainly derived from works which present the results of immunological assays that indicate, overall, an increase in AGE/ALE content caused by the marked oxidative stress base. This work describes for the first time the nature of these adducts and the main plasmatic protein target and opens a new path in the field of HF-characteristic AGE/ALE adduct definition.

This method unequivocally demonstrates a significant increase in CML adducts, the main reaction products between glyoxal and Lys, in the plasma of HF in respect to healthy subjects. Hence, the data firstly indicate an overproduction of glyoxal in HF patients and/or a reduction in its metabolism. Glyoxal is a di-aldehyde which essentially can be generated initially from sugars and lipids (plus their metabolites). Sugars can produce glyoxal through two major pathways: a direct autoxidation reaction, catalyzed by transition metal ions and phosphates, and the Maillard reaction, which generates the Amadori products which are then transformed into glyoxal by enolization. Glyoxal can also be formed from the non-enzymatic peroxidation of polyunsaturated fatty acids, such as linoleic and linolenic acids, forming peroxide intermediates, which then degrade to yield a variety of oxidative products, including glyoxal [9]. By considering that the patients were non-diabetic and the blood glucose as well as the glycated Hb (HbA1C) content did not significantly differ between patients and control subjects, we can reasonably argue that CML adducts arise from lipid peroxidation, the occurrence of which in HF patients is well documented, as recently reviewed by Gianazza et al. [16]. The involvement of GO derived from lipid peroxidation is also evidenced by the fact that the Nε-(Carboxyethyl)-L-lysine (CEL) adducts which are formed from the reaction of Lys residues with methylglyoxal (MGO), a reaction product of glycolysis but not of lipid peroxidation, were not detected. It should be taken into account that albumin from HF subjects is more prone to covalent adduction and this is supported by two results: (1) a significant modification of His residues by iodoacetamide, which, by contrast, was negligible in control subjects and (2) the presence of a deoxy-fructosyl derivative on Lys 389 (DFK-Lys 389) only in HF subjects, despite the fact that the glucose content did not significantly change between the two groups. The alteration of albumin properties in HF and, in particular, its increased susceptibility to covalent adduction is partially supported by the fact that albumin protein conformation, binding activity, and enzymatic activity are greatly affected by covalent adductions as well as by physiopathological conditions such as aging and diseases (e.g., renal or hepatic dysfunction) http://dx.doi.org/10.1016/j.saa.2017.05.023 (accessed on 22 December 2020). 

Considering that CML indicates an increase in lipid peroxidation in HF patients with respect to controls, the question is why other lipid peroxidation adducts were not found to be significantly increased in HF subjects. Although the analytical method was able to detect different lipid peroxidation RCS breakdown albumin adducts such as HNE and ONE in the plasma samples of HF and control subjects, no significant differences were observed. This can be explained by considering different aspects, such as the limited number of subjects and/or the varying metabolic/chemical stability of the identified adducts. The carboxymethyl moiety is an irreversible and relatively stable non-enzymatic modification, at least when compared with Michael adducts formed by α,β-unsaturated aldehydes, which, besides being reversible by a retro-Michael reaction, can undergo further reactions, leading to cross-links which are not detectable by the current analytical approach. Moreover, such adducts can undergo a rapid decarbonylation reaction which, albeit not yet well characterized, has been reported in several conditions [17,18]. Protein decarbonylation and metabolism of albumin adducts in plasma are, to date, an unexplored issue which deserves detailed investigation. Furthermore, ACR adducts were not considered because of their ability of iodoacetamide to react with albumin from HF, forming the carbamidomethylated derivatives whose isotopic mass overlaps with the monoisotopic mass of the ACR adduct in reduced form.

Another issue regards the chemical stability of the adducted proteins during storage. From a chemical point of view, the carboxymethyl moiety should be much more stable in respect to the reversible carbonylated moieties. A possible solution to maintain carbonylated proteins during storage would be to reduce the plasma samples with NaBH_4_ immediately upon blood withdrawal, a procedure which was not applied to the plasma samples analyzed in the present study. Moreover, this aspect deserves further investigation, specifically to design a suitable sample preparation method, which is needed when protein adduction analysis should be carried out.

It is quite clear that more research is needed to understand the metabolic/chemical stability of plasma AGEs/ALEs in order to develop suitable analytical methods and, in particular, sample preparation techniques for their measurement as markers of lipid oxidation. Nonetheless, in the present study, we found that CML is significantly increased in HF subjects and this indicates that, in line of principle, these modifications are stable enough to be used as markers of oxidative stress.

Another important finding of the present paper is that albumin is the main protein target of carbonyls formed in HF. This is not only due to its being the major plasma component but also to the presence of different nucleophilic and exposed residues including Lys, Arg, His, and Cys34 [19]. Hence, we can consider albumin to be a key plasma scavenger of RCS and to be involved in the detoxification reaction of RCS, resulting in endothelial and tissue defense against carbonyl stress.

Albumin has been shown to have an important impact on survival and several papers demonstrate that albumin is an important prognostic parameter of clinical outcome: low serum albumin levels (hypoalbuminemia) are associated with increased risk of HF onset and progression [20]. Several questions arise, particularly regarding the relationship between the reduced amount of blood albumin, which is a clinical feature of HF patients, and RCS levels. In particular, we need to investigate whether albumin reduction is partially due to the albumin RCS modification, which induces a faster catabolism and clearance, as reported for the ACR albumin adduct [21]. Another issue which needs to be investigated is whether albumin reduction in HF patients can compromise the endogenous defense of albumin against RCS stress.

Besides these issues that need to be clarified, we can focus on some key points confirmed by the present study, particularly (i) that HF subjects undergo significant oxidative stress/lipid peroxidation, (ii) that albumin is the main plasma target and the key detoxifier at the extracellular level of RCS, (iii) that GO is formed to a high degree in HF patients, and (iv) that CML is stable enough to be detected as a marker. Besides being a marker of oxidative stress and, in this case, of lipid peroxidation, GO should also be considered as a damaging molecule. GO was found to induce inflammatory injury in human vascular endothelial cells [22], barrier dysfunction, cytoskeletal alterations, and inhibition of angiogenesis in vascular endothelial cells [23]. Hence, a molecular strategy to buffer GO based on maintaining/increasing the endogenous enzymatic and non-enzymatic metabolic defense, such as glutathione (GSH)-dependent metabolism and albumin adduction, should be considered. The recovery of enzymatic metabolic defense of GO and, in general, of lipid-peroxidation-derived RCS can be achieved by replenishing the GSH cellular content which is impaired in CVD conditions and HF [24]. GSH is the nucleophilic substrate of key enzymes for RCS detoxification, such as glyoxalase I and II and GSH transferase. The glyoxalase system is necessary for the metabolic inactivation of di-aldehydes such as glyoxal and methylglyoxal, while GSH-transferase catalyzes the GSH conjugates with α,β-unsaturated aldehydes, which are then detoxified as mercapturic acid derivatives [25]. In the present paper, a significant increase in CML albumin adducts and hence in glyoxal was found, thus suggesting that the glyoxalases’ metabolism is inefficient. N-acetyl cysteine (NAC) is a well-known precursor of GSH and can also act by regenerating Cys34, which is the main circulating antioxidant and scavenger of α,β-unsaturated fatty acids [26,27]. Another approach would consist of maintaining the physiological concentration of circulating albumin by using high-quality albumin infusion. Furthermore, since the above-mentioned enzymes are under stress-responsive control by transcription factor nuclear factor erythroid 2-related factor 2 (Nrf2), activators of Nrf2 could also be effective in reducing RCS-induced damage. Using this approach, recent studies propose that upregulation of Nrf2 attenuates the increase in hemodynamic stress and resultant heart failure [28]. Another interesting approach, which is still in the discovery phase, is based on molecules able to react with carbonyls to form unreactive adducts, thus limiting the carbonylation damage. Much preclinical evidence indicates that compounds effective as RCS-sequestering agents, such as carnosine and derivatives [29,30] and 2-hydroxybenzylamine [31], limit cardiovascular diseases.

From an analytical point of view, the present paper has reported an approach which permits the identification, by an untargeted approach, of albumin covalent modifications induced by lipid-peroxidation-derived RCS. Here, it was applied to HF plasma samples, but, clearly, it can be applied to any plasma samples. Three CML adducts were unequivocally identified and confirmed by standards prepared in in vitro conditions. Furthermore, the CML increase in HF patients was confirmed by an orthogonal validation based on immunological detection. The content of the adducted peptides was here determined as a relative amount in respect to the native peptides; this method permits the measurement and comparison of the relative content of each adduct, hence independently of the absolute amount of albumin, which can vary among individuals and which can significantly differ in pathological conditions. Clearly, the method can be implemented through an absolute quantification approach based on the isotopic internal standard quantification method and on a suitable marker validation according to the current guidelines. Two important aspects to be considered regard the stability of the protein adducts during storage, which needs to be further explored, and the fact that iodoacetamide should not be used as an alkylating agent in studies aimed at searching for ACR adducts.

Although our results were promising, we recognize some limitations. First, this study is limited by the sample size, and it lacks the verification of these adducts on individual samples. Thus, large-scale studies on individuals from various ethnic groups, and with different HF etiology (i.e., ischemic and non-ischemic patients) or differences in the therapy or the presence of other comorbidities, are needed to confirm our results. Second, we did not study HF patients with preserved ejection fraction. Finally, these candidate adducts were assessed at a single time point in stable HF versus healthy volunteers. Therefore, the measurement of their levels, at different time points, to assess their reproducibility in patients in stable conditions or to evaluate the effect of therapies should be performed.

In conclusion, by using an analytical approach, we have unequivocally demonstrated a significant increase in CML albumin adducts in the plasma of HF patients with respect to healthy subjects, indicating an overproduction of glyoxal and or reduction in its metabolic removal in HF patients. Adducts were fully characterized and confirmed by orthogonal approaches. Considering the damaging effect of chronic exposure to GO, molecular strategies aimed at buffering GO and, in line of principle, other RCS, and based on maintaining/increasing the endogenous enzymatic and non-enzymatic metabolic defense, need to be considered. Other lipid peroxidation RCS adducts with albumin were also detected but their amount did not significantly differ in respect to healthy subjects and this could be due to their metabolic and chemical instability, which could affect the quantitative/semi-quantitative analysis. Further studies on this aspect need to be carried out to offer a clear picture of albumin adducts in HF patients, thus providing suitable markers of oxidative and carbonylation damage as well as suitable molecular approaches.

## Figures and Tables

**Table 1:** List of AGE/ALE adducts identified on HSA in control and HF samples and the corresponding aminoacidic sites.

Protein Adducts	Modified AA
Acrolein_Michael_Adduct_NaBH_4_ reduced	His 128, His 242, His 146, His 288, His 510, His 67, Lys 225, Lys 262, Lys 378, Lys 402, Lys 414, Lys 73, His 39
Cysteine_Dioxidation	Cys 245, Cys 246, Cys 34, Cys 392, Cys 487
Cysteine_Trioxidation	Cys 34, Cys 75
Deoxy-fructosyl Derivative	Lys 162, Lys 20, Lys 233, Lys 378, Lys 389, Lys 402, Lys 414, Lys 51, Lys 545, Lys 64, Lys 73
Glyoxal_Carboxymethyl Derivative	Arg 145, Arg 337, Arg 485, Arg 98, Lys 106, Lys 12, Lys 136, Lys 181, Lys 378, Lys 389, Lys 402, Lys 414, Lys 500, Lys 51, Lys 525, Lys 64, Lys 73
Glyoxal_Imidazolone Derivative	Arg 117, Arg 98
4-hydroxy-2-nonenal_Michael Adduct	Lys 41, His 39
Methyl Glyoxal__Carboxyethyl Derivative	Cys 101
4-Oxo-2-nonenal_Lysine_Ketoamide_NaBH_4_ reduced	Lys 402, Lys 41
4-Oxo-2-nonenal_Michael Adduct	Lys 378

**Table 2:** List of the protein adducts identified and the corresponding relative abundance values (%) calculated.

			Relative Abundance (%)	
Peptide Sequence	AGE/ALE Adduct	AA Residue Involved	CTRL	HF
337_RHPDYSVVLLLR_348	1xGO_Carboxymethyl [R1]	Arg 337	0.15	0.13
485_RPCFSALEVDETYVPK_500	1xCarbamidomethyl [C3] 1xGO_Carboxymethyl [R1]	Arg 485	5.81	11.02
11_FKDLGEENFK_20	1xGO_Carboxymethyl [K2]	Lys 12	/	0.44
373_VFDEFKPLVEEPQNLIK_389	1xGO_Carboxymethyl [K6]	Lys 378	/	0.11
373_VFDEFKPLVEEPQNLIKQNCELFEQLGEYK_402	1xCarbamidomethyl [C20]; 1xGO_Carboxymethyl [K6]	Lys 378		
373_VFDEFKPLVEEPQNLIKQNCELFEQLGEYK_402	1xCarbamidomethyl [C20]; 1xGlu_deoxy-fructosil [K17]	Lys 389	/	0.04
390_QNcELFEQLGEYkFQNALLVR_410	C3(Carbamidomethyl); 1xGO_Carboxymethyl [K13]	Lys 402	/	14.76
414_KVPQVSTPTLVEVSR_428	1xGO_Carboxymethyl [K1]	Lys 414	2.45	2.49

**Table 3:** Summary of the results obtained by the manual inspection of the fragmentation profile and the retention time in in-vitro-produced AGE/ALE adducts on HSA, confirming such modifications selected as protein adducts characteristic of the pathological condition (HF). For each sample, the following are reported: (i) the information related to the overlapping fragmentation profile, (ii) the *m*/*z* of the precursor fragmented, and (iii) the retention time, for each adduct searched. n.d. not detected.

*Sample*		CML_Lys 402 (th. *m/z* 886.44637)	CML_Lys 12 (th. *m/z* 428.87506)	CMR_Arg 337 (th. *m/z* 509.28610)	CML_Lys 378 (th. *m/z* 701.70641)
HF_57	Manual check of MS2 Pattern	✓	✓	✓	✓
	Experimental *m/z* (MS2)	886.44953 ± 0.00182	428.87662 ± 0.00073	509.28807 ± 0.00022	701.71150 ± 0.00499
	ppm	−3.55914	−3.85892	−3.85833	−7.25369
	RT (min)	26.27 ± 0.47	10.00 ± 0.03	12.34 ± 0.06	20.44 ± 0.18
HF_114	Manual check of MS2 Pattern	✓	✓	✓	✓
	Experimental *m/z* (MS2)	886.44904 ± 0.00863	428.87747 ± 0.00058	509.28834 ± 0.00074	701.71359 ± 0.00004
	ppm	−3.00638	−5.8758	−4.39829	−10.23209
	RT (min)	25.76 ± 0.11	9.97 ± 0.01	12.26 ± 0.01	20.50 ± 0.54
HSA_GO 1000× 72 h	Manual check of MS2 Pattern	✓	n.d.	n.d.	✓
	Experimental *m/z* (MS2)	886.45081 ± 0.00112			701.71033 ± 0.00604
	ppm	−5.0031			−5.58635
	RT (min)	26.78 ± 0.06			19.94 ± 0.10
Plasma_GO 1000× 48 h	Manual check of MS2 Pattern	n.d.	✓	✓	✓
	Experimental *m/z* (MS2)		428.87727 ± 0.00107	509.28517 ± 0.00073	701.70557 ± 0.00018
	ppm		−5.40947	1.83591	1.20421
	RT (min)		10.35 ± 0.01	13.47 ± 0.29	23.38 ± 0.04
PLASMA_GO 1000× 48 h (reduced by NaBH_4_)	Manual check of MS2 Pattern	n.d.	✓	✓	✓
	Experimental *m/z* (MS2)		428.87649 ± 0.00069	509.28427 ± 0.00076	701.70614 ± 0.00281
	ppm		−3.57912	3.6031	0.38478
	RT (min)		10.32 ± 0.01	13.63 ± 0.04	22.07 ± 0.01
PLASMA_GO 100× 72 h	Manual check of MS2 Pattern	n.d.	✓	✓	✓
	Experimental *m/z* (MS2)		428.87753 ± 0.00033	509.28476 ± 0.00006	701.70514 ± 0.00052
	ppm		−6.0157	2.64096	1.80988
	RT (min)		10.25 ± 0.04	13.47 ± 0.08	23.10 ± 0.06
PLASMA_GO 100× 72 h (reduced by NaBH_4_)	Manual check of MS2 Pattern	n.d.	✓	✓	✓
	Experimental *m/z* (MS2)		428.87665 ± 0.00023	509.2861	701.70590 ± 0.00160
	ppm		−3.95219	5.36047	0.7268
	RT (min)		10.21 ± 0.03	13.7	23.08 ± 0.06
PLASMA_GO 1000× 72 h	Manual check of MS2 Pattern	n.d.	✓	✓	✓
	Experimental *m/z* (MS2)		428.87760 ± 0.00071	509.28570 ± 0.00098	701.70908 ± 0.00142
	ppm		−6.16726	0.78541	−3.79787
	RT (min)		10.17 ± 0.07	13.31 ± 0.01	22.62 ± 0.19
PLASMA_GO 1000× 72 h (reduced by NaBH_4_)	Manual check of MS2 Pattern	n.d.	✓	✓	✓
	Experimental *m/z* (MS2)		428.87624 ± 0.00260	509.28555 ± 0.00024	701.70776 ± 0.00008
	ppm		−2.9962	1.07994	−1.92388
	RT (min)		10.24 ± 0.16	13.40 ± 0.11	22.85 ± 0.16

## Data Availability

The data presented in this study are available on request from the corresponding author.

## Supplementary Materials

The following are available online at https://www.mdpi.com/2076-3921/10/3/358/s1, Table S1. Clinical and demographical characteristics of the study population. Table S2. Individual patients’ demographic data. Table S3. Known (literature-based) covalent adducts induced by HNE, ONE, MDA, ACR, GO, MGO, RED_Sugar derivatives and Cys-oxidation derivatives set as variable modifications within the PD parameters for the identification and localization of AGE/ALE-deriving adducts. Figure S1. Example of the SIC extraction from the total chromatogram (HF 24 sample used for the semi-quantitative analysis of selected adducts. Peptide 287_SH*CIAEVENDEMPADLPSLAADFVESK_313 bearing an ACR-MA on the His 288 residue has been detected in the peptide mixture in two forms: one with oxidized methionine (*m*/*z* 1016.7929) and one without methionine oxidation (*m*/*z* 1011.4601), that were present also for the native peptide (Met_Ox *m*/*z* 997.45205; No_Ox *m*/*z* 992.12015); for the semi-quantitative analysis, all the species mentioned were considered. Other aspecific peaks were detected in the SIC chromatograms of the two modified species: however, only the peaks at 29.17 ± 0.028 min were integrated to calculate the area values since the corresponding MS2 spectra confirmed the peptide sequence; the other peaks probably refer to isobaric species. Table S4. Inclusion List. For each listed adduct are reported: (i) the peptide sequence); (ii) the modification bearing by the peptide; (iii) the aminoacidic site; (iv) the predominant charge state, and the corresponding (v) experimental *m*/*z* of the precursor and (vi) the theoretical one. Figure S2. SDS-PAGE analysis of in vitro produced AGE/ALE adducts by incubating HSA with glyoxal (GO) at increasing molar ratios between protein and GO (1:10, 1:100, 1:1000); the time-dependence effect was also evaluated by stopping the reaction at two time points: after 48 h (panel a) or 72 h (panel b). HSA modified by GO is characterized by a different migration pattern on the gel, with the appearance of oligomeric bands proportional to the increase in the HSA–GO molar ratio.
