# Erratum: Altomare et al. In-Depth AGE and ALE Profiling of Human Albumin in Heart Failure: Ex Vivo Studies. *Antioxidants* 2021, *10*, 358

**DOI:** 10.3390/antiox10101621

**Published:** 2021-10-15

**Authors:** Alessandra Altomare, Giovanna Baron, Marta Balbinot, Alma Martínez Fernández, Alessandro Pedretti, Beatrice Zoanni, Maura Brioschi, Piergiuseppe Agostoni, Marina Carini, Cristina Banfi, Giancarlo Aldini

**Affiliations:** 1 Department of Pharmaceutical Sciences (DISFARM), Università degli Studi di Milano, Via Mangiagalli 25, 20133 Milan, Italy; giovanna.baron@unimi.it (G.B.); marta.balbinot@studenti.unimi.it (M.B.); alma.martinez@uochb.cas.cz (A.M.F.); alessandro.pedretti@unimi.it (A.P.); marina.carini@unimi.it (M.C.); giancarlo.aldini@unimi.it (G.A.); 2 Centro Cardiologico Monzino, IRCCS, Via Parea 4, 20138 Milan, Italy; beatrice.zoanni@ccfm.it (B.Z.); maura.brioschi@cardiologicomonzino.it (M.B.); piergiuseppe.agostoni@cardiologicomonzino.it (P.A.); cristina.banfi@cardiologicomonzino.it (C.B.); 3 Dipartimento di Scienze Cliniche e di Comunità, Sezione Cardiovascolare, Università degli Studi di Milano, Via Festa del Perdono 7, 20122 Milan, Italy

The authors of this paper [1] have agreed they would like to add Alma Martínez Fernández as a co-author, as she contributed to the initial set-up and application of the HRMS untargeted approach in heart failure plasma samples. The authors apologize for not including her in the original paper and for any inconvenience caused to the readers by this change. The updated author contribution is as follows:

Conceptualization, A.A., G.B. and G.A.; Data curation, A.A., G.B., C.B. and G.A.; Formal analysis, A.A., G.B., M.B. (Marta Balbinot), A.M.F., B.Z. and M.B. (Maura Brioschi); Funding acquisition, M.C., C.B. and G.A.; Investigation, A.A., G.B. and A.P.; Methodology, A.A., G.B., M.B. (Marta Balbinot), A.M.F. and M.B. (Maura Brioschi); Project administration, C.B. and G.A.; Resources, M.C., C.B. and G.A.; Supervision, A.A., G.B., C.B. and G.A.; Validation, A.A., G.B. and M.B. (Maura Brioschi); Writing—original draft, A.A., C.B. and G.A.; Writing—review and editing, A.A., M.C., P.A., A.P., C.B. and G.A. All authors have read and agreed to the published version of the manuscript.
