# Supplementary material for: In-Depth AGE and ALE Profiling of Human Albumin in Heart Failure: Ex Vivo Studies
Source: Antioxidants (Basel). 2021 Feb 27;10(3):358. doi: 10.3390/antiox10030358 (PMC7997412; doi:10.3390/antiox10030358)

Supplementary Material

In-depth AGE and ALE profiling of human albumin in Heart Failure: *ex vivo* studies

Alessandra Altomare[1*⊥], Giovanna Baron[1⊥], Marta Balbinot[1], Alessandro Pedretti[1], Marina Carini[1], Beatrice Zoanni[2], Maura Brioschi[2], Piergiuseppe Agostoni[1,3], Cristina Banfi[2] and Giancarlo Aldini[1]

[1] Department of Pharmaceutical Sciences (DISFARM), Università degli Studi di Milano, Via Mangiagalli 25, 20133, Milan, Italy; alessandra.altomare@unimi.it

[2] Centro Cardiologico Monzino, IRCCS, Via Parea 4, 20138, Milan, Italy; Banfi Cristina; cristina.banfi@cardiologicomonzino.it

[3] Dipartimento di Scienze Cliniche e di Comunità, Sezione Cardiovascolare, Università di Milano, Italy. piergiuseppe.agostoni@cardiologicomonzino.it

⊥ These Authors equally contributed to the work

* Correspondence: alessandra.altomare@unimi.it (Department of Pharmaceutical Sciences (DISFARM), Università degli Studi di Milano, Via Mangiagalli 25, 20133, Milan, Italy) (A.A.)

Table S1. Clinical and demographical characteristics of the study population.
* current smokers
DM II, Diabetes mellitus type 2; BMI, body mass index; FE, ejection fraction; BNP, brain natriuretic peptide;

	control subjects		Heart failure patients		
**Characteristics**	*mean*	*SD*	*mean*	*SD*	*pvalue*
Age	53,6	2,1	58,1	6,1	n.s.
Gender (F/M)	2/6		1/9		
Hypertension (n)	0		3		
Dyslipidemia (n)	0		3		
Smoke* (n)	0		1		
DM II (n)	0		3		
BMI (Kg/m^2)	23,5	2,0	26,6	4,0	n.s.
FE (%)	-		30,6	7,5	
Glycemia (mg/dL)	100,6	10,3	118,0	42,2	n.s.

Hb (g/dL)	14,8	0,9	13,5	1,8	n.s
Uric acid (mg/dL)	5,0	0,7	8,1	2,4	0,003
BNP (pg/ml)	-		635,0	462,6	
total cholesterol (mg/dL)	207,6	29,7	183,2	25,4	n.s

Table S2. Known (literature based) covalent adducts induced by HNE, ONE, MDA, ACR, GO, MGO, RED_Sugar derivatives and Cys-oxidation derivatives set as variable modifications within the PD parameters for the identification and localization of AGE/ALE-deriving adducts.

4-hydroxy-2-nonenal (HNE)			
PTM	AA Res. Involved	ΔM (no NaBH4)	ΔM (+ NaBH4)
Michael adduct (HNE-MA)	Cys<br>His<br>Lys<br>Arg	+ 156.110502 Da	+ 158.13067 Da
Schiff Base (HNE-SB)	Lys<br>Arg	+ 138.01446 Da	+ 140.12011 Da
2-pentilpyrrole (PP)	Lys<br>Arg	+ 120.0939 Da	-
Dehydropentylfuran (DHPF)	Cys<br>His<br>Lys<br>Arg	+ 138.10446 Da	-
**4-oxo-2-nonenal (ONE)**			
PTM	AA Res. Involved	ΔM (no NaBH4)	ΔM (+ NaBH4)
Michael adduct (ONE-MA)	Cys<br>His<br>Lys<br>Arg	+ 154.09937 Da	+ 158.13067 Da
Schiff Base (ONE-SB)	Lys<br>Arg	+ 136.08881 Da	+ 140.12011 Da
ONE-pentylfuran (OPF)	Cys<br>His<br>Lys<br>Arg	+ 136.08881 Da	-
**Malondialdehyde (MDA)**			
PTM	AA Res. Involved	ΔM (no NaBH4)	ΔM (+ NaBH4)

N-propenal-Lysine (NPK)	Lys	+ 54.01056 Da	+ 56.02621 Da
Di-dihydropyridine-lysine (DHPK)	Lys	+ 134.03678 Da	+ 138.06808 Da
Malondialdehyde argpyrimidine(MDA-RP)	Arg	+ 36.0000 Da	-
**Acrolein (ACR)**			
PTM	AA Res. Involved	ΔM (no NaBH4)	ΔM (+ NaBH4)
Michael Adduct (propanal derivative) (ACR-MA)	Cys<br>Lys<br>His	+ 56.02621 Da	+ 58.04186 Da
Hydroxy-tetra-hydropyrimidine (Propane-arginine) (HTPR) (HTPR)	Arg	+ 56.02621 Da	-
Double Michael Adduct (2ACR-KMA)	Lys	+ 112.05243 Da	+ 116.08373 Da
Schiff Base (ACR-SB)	Lys	+ 38.01565 Da	+ 40.0313 Da
Formyl-dehydro-piperidyl-lysine (FDPK)	Lys	+ 94.04186 Da	+ 96.05751 Da
Methylpyridine-lysine (MPK)	Lys	+ 77.03912 Da	-
**Glyoxal (GO)**			
PTM	AA Res. Involved	ΔM (no NaBH4)	*ΔM (+ NaBH4)*
Carboxymethil derivative (GO-CM)	Lys<br>Arg	+ 58.005479 Da	-
Glyoxal Imidazolone (GO_IR)	Arg	+ 39.99492	-
**Methyl Glyoxal (MGO)**			
PTM	AA Res. Involved	ΔM (no NaBH4)	*ΔM (+ NaBH4)*
Carboxyethil derivative (MGO-CE)	Cys<br>Lys<br>Arg	+ 72.02113 Da	-
Methylglyoxal Methylimidazolone Derivative (MGO-RMI)	Arg	+ 54.01056 Da	-
Methylglyoxal Pirimidine Derivative (MGO-RP)	Arg	+ 80.02622 Da	-
Methylglyoxal Tetra Hydro Pirimidine Derivative (MGO-THPR)	Arg	+ 144.04226 Da	-
**Sugar Derivatives**			
PTM	AA Res. Involved	ΔM (no NaBH4)	*ΔM (+ NaBH4)*
Glucose Deoxy-fructosil Deirvative (DFK/DFR)	Lys<br>Arg	+ 162.05282 Da	-
3-Deoxyglucosone-Imidazolone Derivative (3DG-IR)	Arg	+ 144.04226 Da	-
*Glucose Pirraline derivative (*PK*)*	Lys	+ 108.1723 Da	-
**Cysteine-Oxo Derivatives**			
PTM	AA Res. Involved	ΔM (no NaBH4)	*ΔM (+ NaBH4)*
Sulfenic Acid-Cys (Sulfe_AC)	Cys	+ 15.99492 Da	-

Sulfinic Acid-Cys (Sulfi_AC)	Cys	+ 31.98983 Da	
Sulfonic Acid-Cys (Sulfo_AC)	Cys	+ 47.98474 Da	
Glutathione Disulfide Adduct (GSS-)	Cys	+ 305.681596 Da	-
S-Nitrosilation (S-Nicro_Cys)	Cys	+ 28.99016 Da	-

Figure S1. Example of the SIC extraction from the total chromatogram (HF 24 sample used for the semi-quantitative analysis of selected adducts. Peptide 287_SH*CIAEVENDEMPADLPSLAADFVESK_313 bearing an ACR-MA on the His 288 residue has been detected in the peptide mixture in two forms: one with oxidized methionine (m/z 1016.7929) and one without methionine oxidation (m/z 1011.4601), that were present also for the native peptide (Met_Ox m/z 997.45205; No_Ox m/z 992.12015); for the semi-quantitative analysis, all the species mentioned were considered. Other aspecific peaks were detected in the SIC chromatograms of the two modified species: however, only the peaks at 29.17 ± 0.028 min were integrated to calculate the area values since the corresponding MS2 spectra confirmed the peptide sequence; the other peaks probably refer to isobaric species.

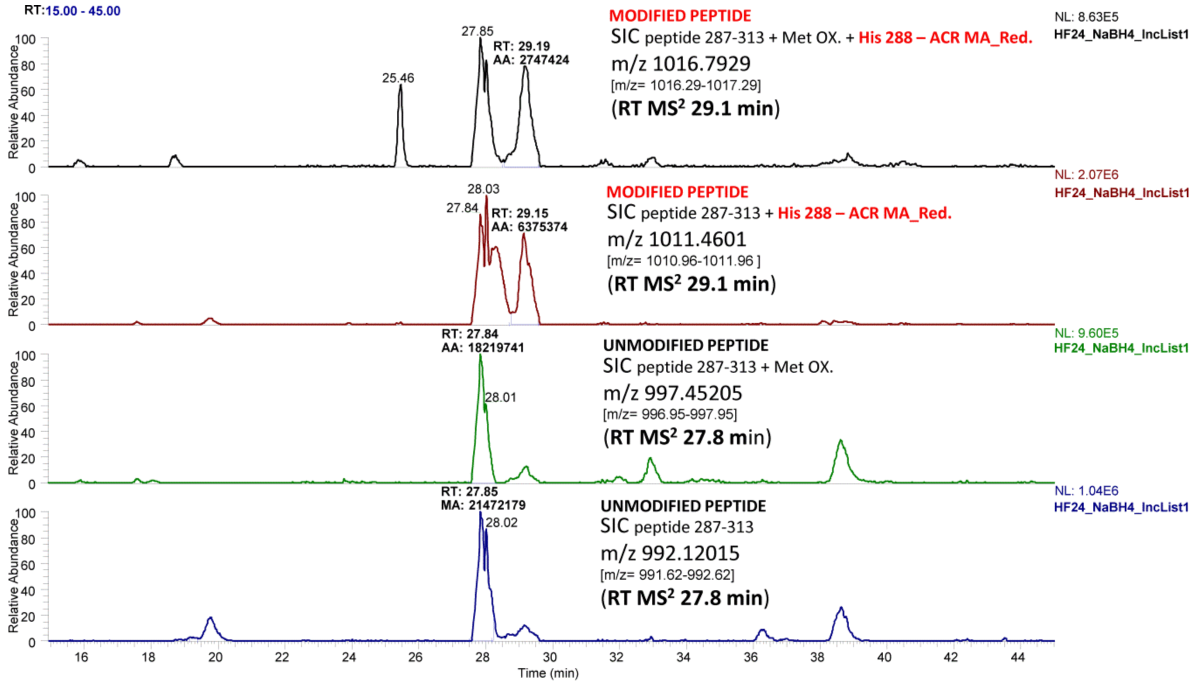

$$\boldsymbol{Add.Rel.Ab.}(\%) = \left[\frac{\frac{Mod.Peptide}{Peak\ Area}}{\left(\frac{Mod.Peptide}{Peak\ Area+UnMod.Peptide}\right)\atop Peak\ Area}\right] * 100 = \left[\frac{(2747424+6375374)}{(2747424+6375374+18219741+2142179)}\right] * 100 = 18.688\%$$

Table S3. Inclusion List. For each listed adduct are reported: (i) the peptide sequence); (ii) the modification bearing by the peptide; (iii) the aminoacidic site; (iv) the predominant charge state, and the corresponding (v) experimental m/z of the precursor and (vi) the theoretical one.

Peptide Sequence	AGE/ALE Adduct	AA residue involved	Charge	m/z Sp.	m/z th. (Inclusion list)
337_RHPDYSVVLLLR_348	1xGO_Carboxymethyl [R1]	Arg 337	3	509,6208	509,2861
337_RHPDYSVVLLLR_348	-	-	3	489,9513	489,95249
485_RPCFSALEVDETYVPK_500	1xCarbamidomethyl [C3]<br>1xGO_Carboxymethyl [R1]	Arg 485	3	657,3184	657,3184
485_RPCFSALEVDETYVPK_500	1xCarbamidomethyl [C3]	-	3	637,982	637,982
11_FKDLGEENFK_20	1xGO_Carboxymethyl [K2]	Lys 12	3	428,87495	428,87495
11_FKDLGEENFK_20	-	-	3	409,5388	409,5388
373_VFDEFKPLVEEPQNLIK_389	1xGO_Carboxymethyl [K6]	Lys 378	3	702,0385	702,0385
373_VFDEFKPLVEEPQNLIKQNCELFEQLGEYK_402	1xCarbamidomethyl [C20];<br>1xGO_Carboxymethyl [K6]	Lys 378	3	1247,5939	1247,95628
373_VFDEFKPLVEEPQNLIKQNCELFEQLGEYK_402	1xCarbamidomethyl [C20];<br>1xGlu_deoxy-fructosil [K17]	Lys 389	4	962,4763	962,4763
373_VFDEFKPLVEEPQNLIK_389	-	-	3	682,7026	682,37068
390_QNcELFEQLGEYkFQNALLVR_410	C3(Carbamidomethyl);<br>1xGO_Carboxymethyl [K13]	Lys 402	3	886,7759	886,44583
390_QNcELFEQLGEYKFQNALLVR_410	C3(Carbamidomethyl);	-	3	867,4366	867,10416
414_KVPQVSTPTLVEVSR_428	1xGO_Carboxymethyl [K1]	Lys 414	3	566,9925	566,65944
414_KVPQVSTPTLVEVSR_428	-	-	3	547,3155	547,31749

Figure S2. SDS-PAGE analysis of *in vitro* produced AGEs/ALEs adducts by incubating HSA with glyoxal (GO) at increasing molar ratios between protein and GO (1:10, 1:100, 1:1000); the time-dependence effect was also evaluated stopping the reaction at two time points: after 48 h (*panel a*) or 72 h (*panel b*). HSA modified by GO is characterized by a different migration pattern on the gel, with the appearance of oligomeric bands proportional to the increase of the HSA-GO molar ratio.

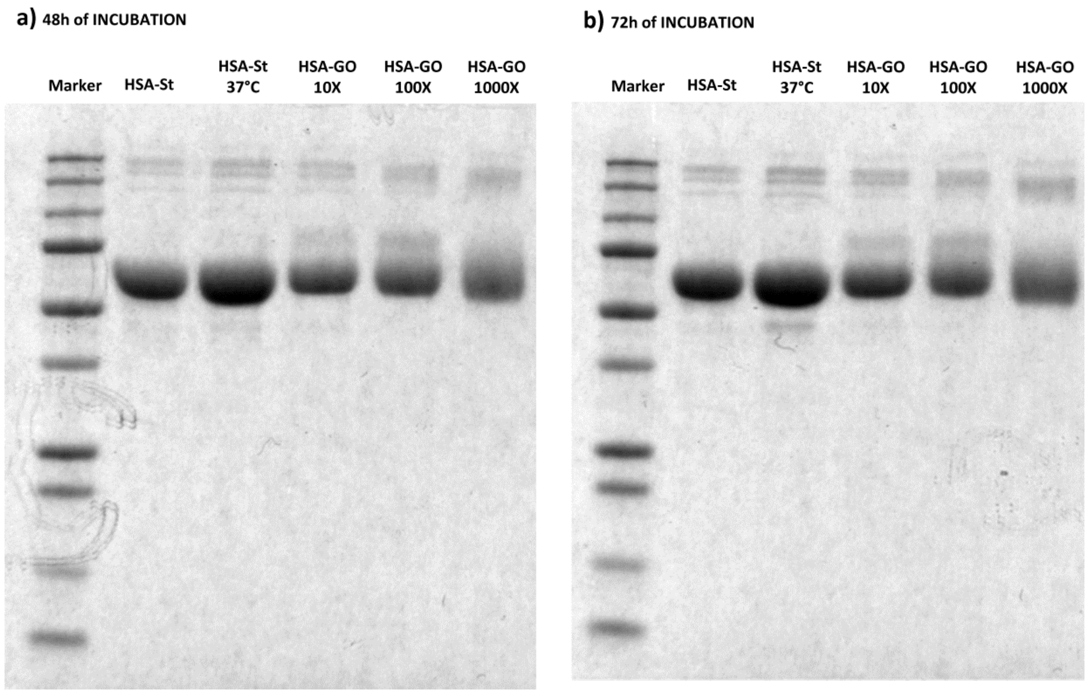

Supplement: Supplementary file 1 [file antioxidants-10-00358-s001.pdf]
